# Somatosensory evoked potentials and their relation to microstructural damage in patients with multiple sclerosis—A whole brain DTI study

**DOI:** 10.3389/fneur.2022.890841

**Published:** 2022-08-29

**Authors:** Jan Hamann, Barbara Ettrich, Karl Titus Hoffman, Florian Then Bergh, Donald Lobsien

**Affiliations:** ^1^Institute of Neuroradiology, University of Leipzig, Leipzig, Germany; ^2^Department of Neurology, University of Leipzig, Leipzig, Germany; ^3^Institute of Diagnostic and Interventional Radiology and Neuroradiology, Helios Klinikum Erfurt, Erfurt, Germany

**Keywords:** diffusion tensor imaging (DTI), MRI, somatosensory evoked potential (SSEP), multiple sclerosis, sensible tract

## Abstract

**Introduction:**

Somatosensory evoked potentials (SSEP) play a pivotal role in the diagnosis and disease monitoring of multiple sclerosis (MS). Delayed latencies are a surrogate for demyelination along the sensory afference. This study aimed to evaluate if SSEP latencies are representative of demyelination of the brain overall, by correlating with cerebral microstructural integrity as measured by Magnetic resonance (MR) diffusion tensor imaging (DTI). Analysis was performed in a hypothesis-free whole brain approach using tract-based spatial statistics (TBSS).

**Material and methods:**

A total of 46 patients with MS or clinically isolated syndrome were included in the study. Bilateral SSEPs of the median nerve measuring mean N20 latencies (mN20) and Central Conduction Time (CCT), were acquired. MRI scans were performed at 3T. DTI acquisition was done with a single-shot echoplanar imaging technique with 80 diffusion directions. The FSL software package was used to process the DTI datasets and to calculate maps of fractional anisotropy (FA), axial diffusivity (AD), and radial diffusivity (RD). These maps were then further analyzed using the TBSS module. The mean N20 and CCT and the right- and left-sided N20 and CCT were separately correlated to FA, AD, and RD, controlled for age, gender, and EDSS as variables of non-interest.

**Results:**

Widespread negative correlations of SSEP latencies with FA (*p* = 0.0005) and positive correlations with RD (*p* = 0.0003) were measured in distinct white matter tracts, especially the optic tracts, corpus callosum, and posterior corona radiata. No correlation with AD was found in any white matter tract.

**Conclusion:**

Highly significant correlations of FA and RD to SSEPs suggest that their latency is representative of widespread microstructural change, and especially demyelination in patients suffering from MS, reaching beyond the classic somatosensory regions. This points to the usefulness of SSEPs as a non-invasive tool in the evaluation of microstructural damage to the brain.

## Introduction

White matter is the primary location of brain damage in patients suffering from multiple sclerosis (MS). The primary imaging modality to evaluate this damage is magnetic resonance imaging (MRI) (1, 2). Conventional sequences such as FLAIR, T2, and T1 weighted sequences with or without contrast agents are the mainstay of routine diagnostic imaging. However, microstructural analysis is beyond the scope of these imaging techniques. Therefore, substantial effort is put into the research and development of microstructural imaging, which offers further insight into the analysis of the so-called normal appearing white matter as well. One of these techniques is diffusion tensor imaging (DTI), which allows microstructural brain imaging by measuring the directional movement of water molecules (3). DTI provides various parameters to allow further insight into microstructural brain damage, beyond that detected by standard diagnostic MRI. Three of these parameters are fractional anisotropy (FA), radial diffusivity (RD), and axial diffusivity (AD). One elaborate way is to analyze the data provided by DTI and these parameters with tract-based spatial statistic imaging (TBSS), provided in the freely available FSL imaging analysis package (FMRIB, Oxford, Great Britain). With TBSS, a voxel wise statistical analysis of whole brain diffusion datasets can be performed (4, 5). For example, hypothesis-free whole brain voxel-wise analysis of correlations of microstructural characteristics with functional testing is possible (6).

Previously, we have shown that microstructural alterations of white matter tracts as detected by DTI and TBSS show widespread associated damage to the visual system as measured by visual evoked potential (VEP) latencies in patients suffering from MS (7). These alterations did not only affect the visual system pathways but also other parts of the brain such as the corpus callosum, with or without detectable lesions on conventional MR-imaging, indicating that VEPs are also a measure of the damage to the central nervous system (CNS) in general. Another system of crucial importance is the somatosensory system which is frequently affected in MS. Therefore, an analysis of its relation to microstructural damage in patients with MS seems warranted. Furthermore, SSEPs are a routinely applied electrophysiological test in patients with MS, increasing the interest in its association with microstructural CNS damage.

## Materials and methods

We retrospectively analyzed MRI datasets and SSEP measurements in conjunction with clinical parameters of patients suffering from MS collected in a prospectively kept database. The study was approved by the ethics committee of the University of Leipzig, Medical Faculty (130/2007).

### Patients

We included patients with clinically definite MS and clinically isolated syndromes (CIS) who were screened for an investigator-initiated trial studying the potential remyelinating activity of rhGH (EudraCT 2006-006465-16). All patients underwent the same procedure of neurological and electrophysiological examination, MRI acquisition was performed at 3T, including DTI and standard diagnostic sequences.

### Patient inclusion criteria

– Patients older than 18 years suffering from MS [relapsing-remitting MS (RRMS) or primary progressive (PPMS)] or a clinically isolated syndrome.
– Complete 3T MRI including a DTI-dataset as specified under imaging.
– Complete diagnostic SSEP examination within a timeframe of 3 months in relation to the timing of the MRI.
– Clinical examination including expanded disability scale scoring (EDSS) within a timeframe of 3 months in relation to the MRI.

### Patient exclusion criteria

– Acute relapse as defined by clinical criteria during the study period and within 6 months before the first examination.

Treatment of the patients with disease-modifying drugs was not an inclusion or exclusion criterion, i.e., patients with or without treatment could be included.

### Somatosensory evoked potentials

Somatosensory evoked potentials were measured in a standard clinical fashion according to the guidelines of the German Society for Clinical Neurophysiology (DGKN), using the same stimulation and recording apparatus (Nihon Koden) in the same neurophysiology lab for all measurements. SSEPs for the upper limbs were obtained by using electrical stimulation (duration 0.1 ms, frequency 3.9 Hz) of the median nerve at the wrist. The main latencies measured were at Erb's point (N9), lower and upper cervical spine (N13a and N13b) and at the contralateral primary sensory cortex (N20). We calculated the Central Conduction Time (CCT) subtracting N20-N13b (N20 upper limit of normal (ULN) 22.0 ms, CCT ULN 7.7 ms). In our analysis, we considered the N20 and CCT latencies for the correlation. The average of the measured latencies and calculated intervals of both sides and the right- and left-sided latencies served as the input variable for TBSS analysis.

### MRI

All MRI scans were performed on a 3T MR imaging scanner (Magnetom Trio; Siemens, Erlangen, Germany) using a 12-channel head coil. The imaging protocol included a Fluid Attenuated Inversion Recovery sequence (FLAIR), T1-weighted sequences with and without a standard dose of gadolinium contrast agent, T2 weighted sequences in axial and sagittal planes. A DTI dataset was acquired with the following parameters: single shot echo planar, TR 2,700ms, TE 93 ms; flip angle 90°; parallel imaging, 80 diffusion-encoding gradient directions, 1 B0 image; b 1,000 s /mm^2^, matrix size 128 × 128; voxel size 1.8 mm × 1.8 mm × 5 mm.

### Diffusion tensor imaging

Diffusion tensor imaging workflow and TBSS pipeline were carried out according to the method described previously (7). All image processing was performed in the FSL software package (FMRIB [FSL; http://www.fmrib.ox.ac.uk/fsl]). Preprocessing steps were: (1) Brain extraction by using FSL's brain extraction tool (8). (2) Correction for subjects moving and eddy current artifacts using eddy_correct (9). (3) Calculation of FA, AD, and RD from the Eigenvalues using DTIFit.

Then TBSS was used for the voxel based investigation (5). First, all FA data were aligned to 1 mm × 1 mm × 1 mm standard space using the non-linear registration tool (FNIRT) and averaged (10, 11). The mean FA image was subsequently skeletonized (tbbs_3_postreg) and thresholded at 0.2 to exclude peripheral tracts and gray matter. Afterward, each FA image was projected onto the mean FA-skeleton common to all subjects. The AD and RD data were processed similarly, by using the FA-skeleton of the previous steps.

Statistics were done with the Randomize tool, a part of the FSL package. Randomize combines a General Linear Model (GLM) matrix and permutation test theory (12). For the GLM, we used mN20, mCCT, and the recorded right- and left-sides latencies as a continuous variable of interest and correlated with the diffusion parameters FA, AD, and RD, controlled for age, gender, and EDSS score as covariates of non-interest. All data were demeaned. The threshold-free cluster enhancement method was used to enhance cluster-like structures. Subjects were examined with 10.000 permutations with the maximum threshold free-cluster enhancement recorded at each permutation, corrected for multiple comparisons by controlling for family-wise error rates (13). *p* < 0.05 was used as a threshold for statistical significance.

### Region of interest analysis

**Figure 1:**
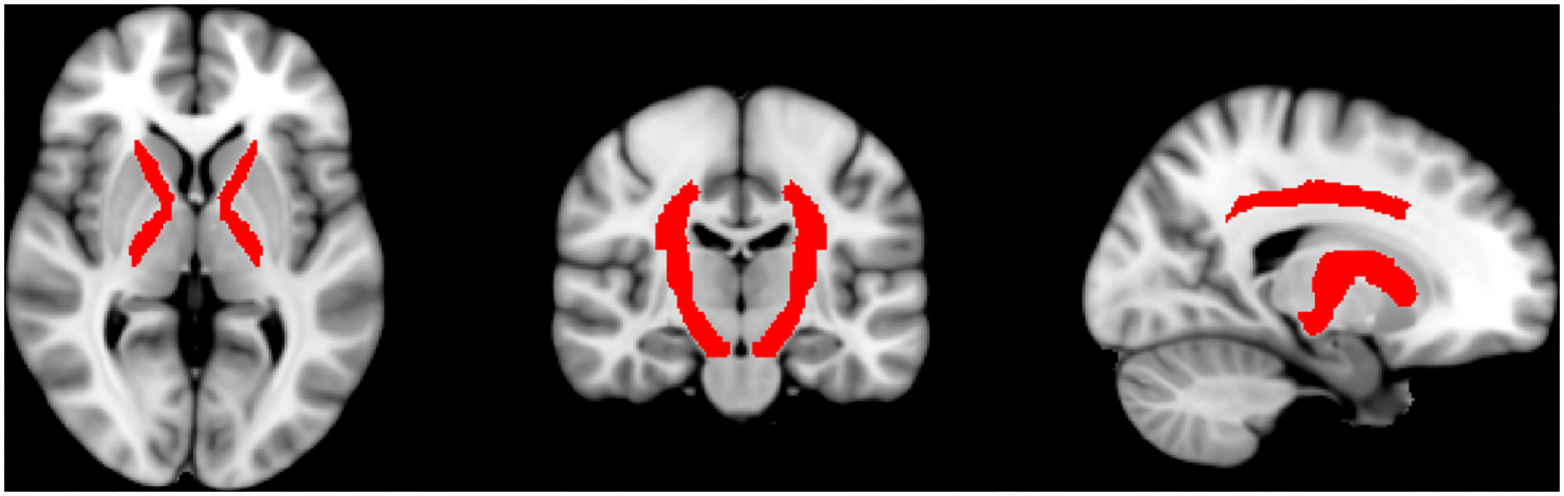
Shown are the automatically drawn ROIs that correspond to sensory tracts by using the DTI-81 Atlas in the FMRIB library package.

Supplementary to that, we evaluated multiple regions of interest (ROIs) covering areas with special relevance to the somatosensory system and regions with high-significant regions and wide clusters. For this, we used the JHU ICBM DTI-81WM-Labels Atlas, which can be freely downloaded and used within the FSL software package (http://www.loni.usc.edu/ICBM/Downloads/Downloads_DTI-81.shtml) (14). The regions that should be included were selected manually and subsequently, the exact size of the ROIs was automatically defined on the co-registered brains according to the defined size of the regions of the atlas. The ROIs corresponding to somatosensory regions included the anterior limb of the internal capsule (ALIC), posterior limb of the internal capsule (PLIC), corona radiata superior (CRS), and posterior (CRP) and the cerebral peduncles. Further ROIs were the body, genu, and splenium of the corpus callosum, post-thalamic radiation (includes optic radiation), and superior longitudinal fasciculus. A graphic representation of the sensory ROIs is shown in Figure 1. By using the above-drawn masks, the significant (*p* ≤ 0.05) FA and RD values for each subject were extracted by using fslmaths and flsmeants. Then, FA, RD, and SSEPs were correlated by using Spearman correlation with a correction for multiple comparisons to prevent type 1 error by using Bonferroni correction. Prism 9 Software (Graphpad, San Diego, CA, USA) was used to perform these correlations. Classification of correlation coefficient rho is low (0.0–0.2), moderate (0.2–0.5), high (0.5–0.8), and perfect (0.8-1.0).

### Quantification of significant voxels

To evaluate and quantify the asymmetry of significant voxels with *p* ≤ 0.05 we analyzed the absolute amount in the GLM-results for mean, right-/left-sided N20, and CCT. For this, we calculated homologous masks using fslmaths that corresponded exactly to the right hemisphere and left hemisphere. With fslstats, significant voxels were counted in both hemispheres separately for FA and RD. An intensive focus was assessed on asymmetry in our sensory ROIs, defined above.

### Lesion mapping

To evaluate the anatomical relationship of white matter lesions and normal appearing white matter to the correlations found between DTI and SSEPs, lesion mapping was performed according to the method described in our previous articles (7). First, the FLAIR datasets were co-registered to 1 mm × 1 mm × 1 mm standard space (MNI 152). Then images were manually segmented and binary masks were drawn by hand, using FSLeyes (https://fsl.fmrib.ox.ac.uk/fsl/fslwiki/FSLeyes). The resulting masks included the MS lesions of each subject. By using fslmaths, we summarized these masks and plotted them with a colorized scale to indicate regions with a high or low incidence of lesions among the subjects. We merged the resulting map with the results of tract-based spatial statistics analysis to qualitatively visualize lesions in relationship to the correlations.

## Results

### Patients

A total of 46 patients were included in this study, 38 of them with clinically definite MS (37 RRMS, 1 PPMS) and 8 with a clinically isolated syndrome (CIS 8). The included patients were aged between 18 and 67 years. The median EDSS was 2.0. Twenty-four patients were on disease-modifying drugs. No gadolinium-enhancing lesions on T1-weighted images were detected after contrast administration. Median N20 was 20.03 ms and mCCT was 6.39. According to ULN specifically established for the Department of Neurology electrophysiology lab, University of Leipzig, pathologically delayed N20 latencies were detected in 6/46 patients (13.0%), Whereas, 4/46 subjects (8.7%) had pathologically delayed CCT latencies. Disease duration time could be reported for 36 subjects. Additional clinical data are shown in Table 1.

**Table 1:** Clinical characteristics of the patients.

Patients	46 (28 female, 18 male)
Age [years, mean + −SD]	38,2 + −12,02
**Diagnosis**	
CIS	8
RRMS	37
PPMS	1
EDSS [median/IQR]	2.0 /3.0
mN20 [median/IQR]	20.03/1.74 (ULN 22.0 ms)
right-side N20 [median/IQR]	20.25/1.62
left-side N20 [median/IQR]	19.88/2.01
Pathological N20	6 subjects (3 with both sides, 1 right, 2 left)
N20 interside difference [median/IQR]	0.48/0.56 (ULN 1.20)
Pathologic interside difference	7 subjects
mCCT [median/IQR]	6.39/1.02 (ULN 7.7 ms)
right-side CCT [median/IQR]	6.35/0.99
left-side CCT [median/IQR]	6.35/1.02
Patholgical CCT	4 subjects (2 with both sides, 1 right, 1 left)
CCT interside difference [median/IQR]	0.35/0.51 (ULN 1.35)
Pathologic interside difference	6 subjects

The majority of the patients suffered from RRMS were female. The overall severity of the illness was moderate as measured by the EDSS scale.

CIS, Clinical isolated syndrome; RRMS, relapsing-remitting MS; PPMS, Primary Progressive MS; EDSS, Expanded Disability Status Scale; mCCT, mean Central conducting time; ULN, Upper Limit Norm corresponding to the Department of Neurology electrophysiology lab, University of Leipzig.

### Whole brain fractional anisotropy

We found highly significant negative, moderate to high correlations (rho −0,54, *p* 0.005) between mN20 latencies and FA in widespread regions of the brain. The most significant findings (up to *p* ≤ 0.01) were located in the frontotemporal white matter, corona radiata posterior (rho −0.39/*p* 0.008) et superior (rho −0.36/*p* 0.02) bitemporally, genu (rho −0.42/*p* 0.004) and splenium(rho −0.4/*p* 0.007) of the corpus callosum, the post-thalamic radiation (including optic radiations) of both sides (rho −0.4/*p* 0.005), and the superior longitudinal fasciculus (rho −0.46/*p* 0.001). Findings with a significance level up to *p* ≤ 0.05 and moderate correlation were found in multiple locations along the skeleton, including the anterior corona radiata and posterior internal capsule on the right side (rho −0.33/*p* 0.025). Asymmetry was found in the internal capsule with no significant voxels on the left side. No correlation was found between cerebral peduncles and the anterior limb of the internal capsule.

**Figure 2:**
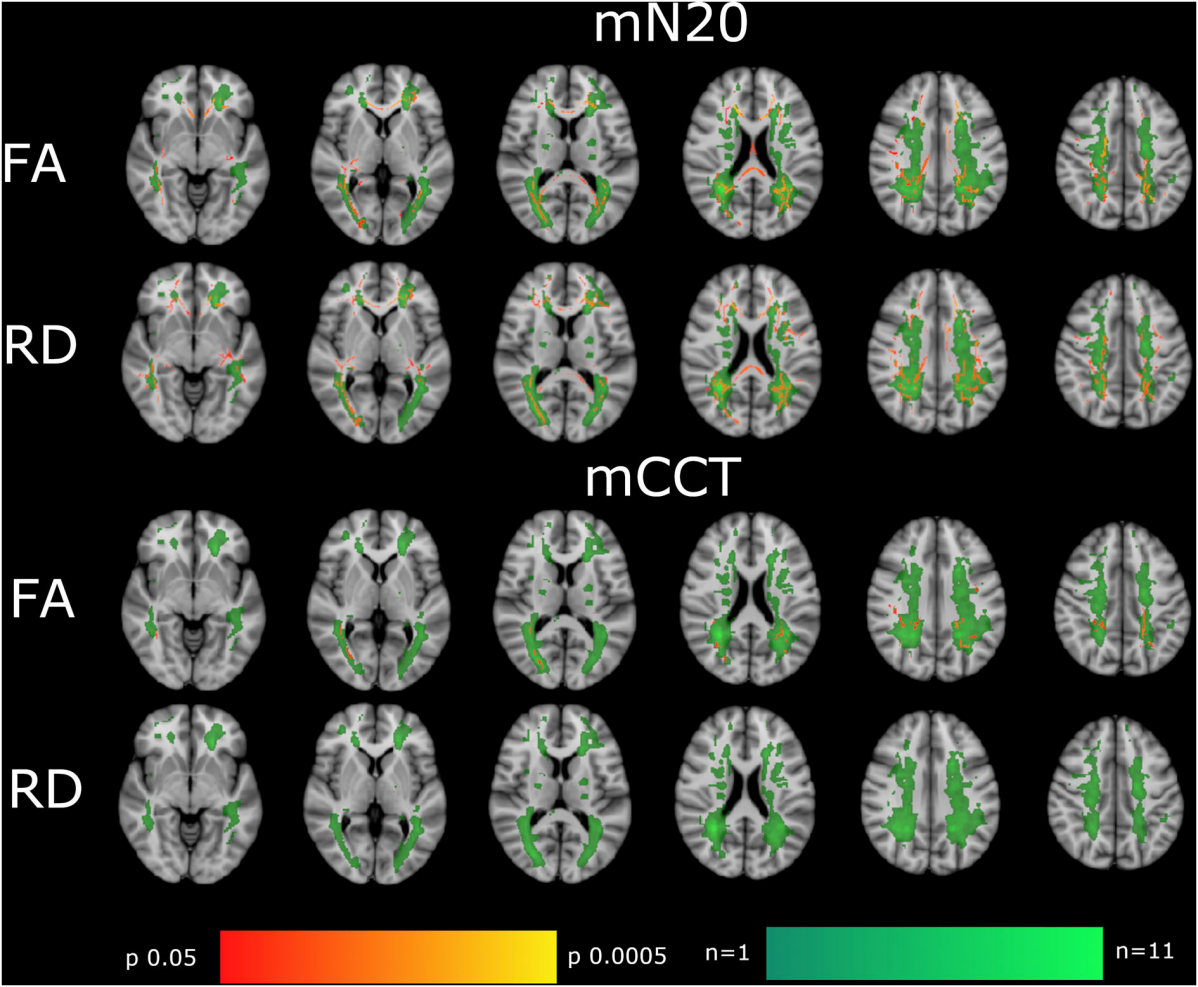
Shown are the correlations of FA, RD with mN20, and mCCT of the tract-based spatial statistics results based on the GLM-model. Significant correlations are highlighted from red to yellow, from *p* = 0.0005 to *p* = 0.004. To visualize MS lesions the summarized lesions maps are presented in green. Dark green indicates regions where only 1 patient had a lesion, Whereas, light green indicates regions where 11 patients had lesions. The T1 Montreal Neurological Institute 158 brain is the background image. First row: correlation of FA with mN20. Second row: RD with mN20. Third row: FA with mCCT. Fourth row: RD with mCCT. Correlations are widespread in the brain with the highest correlation in the Corona radiata superior and posterior, the visual tract and corpus callosum. MS lesions are most frequently around the ventricles. Locations with the most lesions are parieto-occipital.

Mean CCT intervals also showed significant negative, moderate to high correlations to FA (rho −0.56/*p* 0.0005), even though with less significant regions and smaller clusters compared to mN20 latencies. Regions with a significance level up to *p* ≤ 0.01 and moderate correlation were found in the temporal white matter (with a slight left predominance), corona radiata posterior bitemporally (rho −0.48/*p* 0.0007), corona radiata superior of the left side (rho −0.4/*p* 0.005), splenium of the corpus callosum (rho −0.37/*p* 0.01). Highest significant correlations up to *p* ≤ 0.0001 were found in the posterior corona radiata and the superior longitudinal fasciculus of the left side (rho −0.48/*p* 0.0007). More details are shown in Figure 2.

### Whole brain radial diffusivity

Significant positive, moderate to high correlations were found between RD and mN20 along the skeleton (rho 0.51/*p* 0.0003). Highest significance levels up to p ≤ 0.01 were found in the corona radiata posterior bilaterally (rho 0.42/*p* 0.004) and superior (rho 0.38/*p* 0.009) of the left side, in the corpus callosum (rho 0.5/*p* 0.0004) and the post-thalamic radiation (including optic radiations)(rho 0.48/*p* 0.0007). In the superior longitudinal fasciculus of the left side (rho 0.34/*p* 0.02) and the frontotemporal white matter, we found correlations up to *p* ≤ 0.05 with moderate correlation as well. No correlations between RD and mCCT were found. Down to a significance level of *p* = 0.08, clusters in the posterior corona radiata and superior longitudinal fasciculus can be reported.

### Whole brain axial diffusivity

Axial Diffusivity showed no significant correlations with mN20, mCCT, right- and left-sided N20, and CCT.

### The difference in the right- and left-sided N20 and CCT

N20 latencies from the right and left sides showed a significant moderate to high correlation to FA and RD in a very different pattern. Whereas, left-sided N20 correlates in comparable regions as described in the mN20 paragraph to FA and RD, but with higher significance levels, right-sided N20 correlates less than mN20 to FA and does not correlate to RD. It is remarkable that left-sided N20 shows larger clusters in more regions correlating to FA and RD compared to mN20 and right-sided N20. Particularly, sensory regions in the right hemisphere show stronger relation to left-sided N20. For example, the posterior limb of the internal capsule of the right side shows a significant negative moderate correlation to FA (rho −0.4/*p* 0.006), Whereas, the left side with lower correlation (rho −0.33/*p* 0.025) and posterior corona radiata of the right side (rho −0.4/*p* 0.006) and left side (rho −0.36/*p* 0.013). A similar situation is seen in right-sided N20: superior corona radiata of the left side correlates significantly and negatively with FA (rho −0.36/*p* 0.015), Whereas, no correlation on the right side is found.

**Figure 3:**
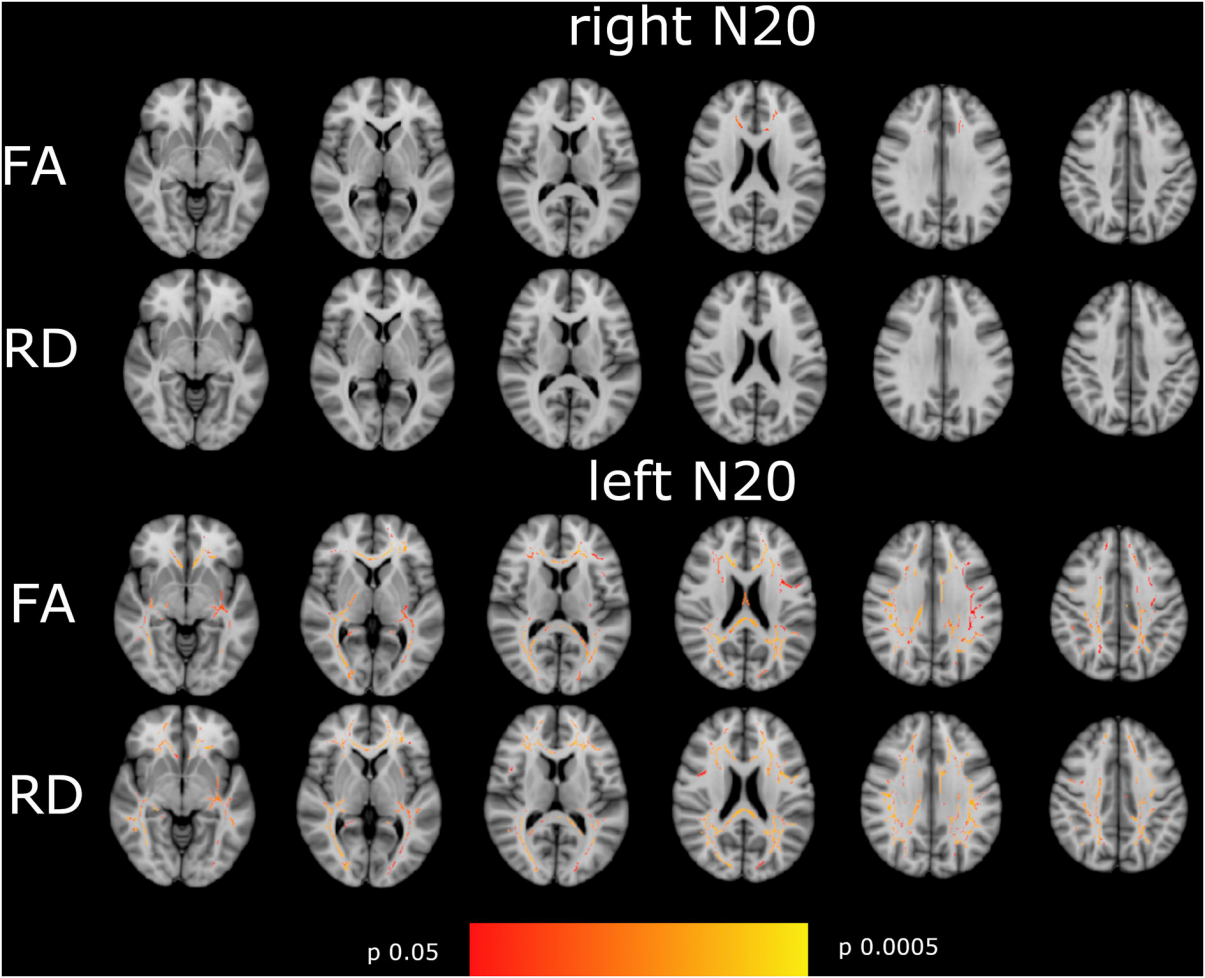
Shown are the correlations of FA, and RD with right- and left-sided N20 of the tract-based spatial statistics results based on the GLM-model. Significant correlations are highlighted from red to yellow, from *p* = 0.0005 to *p* = 0.004. The T1 Montreal Neurological Institute 158 brain is the background image. First row: correlation of FA with right-sided N20. Second row: RD with right-sided N20. Third row: FA with left-sided N20. Fourth row: RD with left-sided N20. Correlations are widespread in the brain with the highest correlation in the corona radiata superior and posterior, the visual tract, and the corpus callosum in left-sided N20. Hardly correlations were found in right-sided N20 except for corpus callosum. To focus on side differences lesions are not shown.

In remarkable contrast to the findings in N20 latencies, right- and left-sided CCT show different appearances: no correlation, whether with FA or RD, was found with sided CCT. It is worth mentioning that left-sided CCT with a significance level down to *p* = 0.06 shows a correlation to RD in corona radiata posterior et superior, post-thalamic radiation, and superior longitudinal fasciculus predominantly on the right side. More details are shown in Figure 3, Tables 2, 3.

**Table 2:** Shown are the Spearman-Correlation values of N20-latencies from the right and left median nerve for selected ROIs and the DTI-derived parameter Fractional Anisotropie (FA).

**ROI**	**Right-side-N20—FA (rho/*p*-value)**			**Left-side-N20—FA (rho/*p*-value)**		
Whole Brain	−0.45/0.0001			−0.52/0.0002		
Sensible-Tract	−0.36/0.015			−0.4/0.006		
Body CC	−0.39/0.007			−0.47/0.001		
Genu CC	−0.38/0.01			−0.4/0.005		
Splenium CC	*			−0.37/0.01		
	both	right	left	both	right	left
ALIC	*	*	*	*	*	*
Cerebral Peduncle	*	*	*	*	*	*
PLIC	*	*	*	−0.4/0.005	−0.4/0.006	−0.33/0.025
Corona radiata post.	*	*	*	−0.37/0.01	−0.4/0.006	−0.36/0.013
Postthalamic radiation	*	*	*	−0.42/0.004	−0.39/0.008	−0.45/0.001
Corona radiata superior	*	*	−0.36/0.015	−0.36/0.015	−0.36/0.014	−0.37/0.01
Superior longituidinal fasciculus	*	*	*	−0.37/0.01	−0.36/0.012	−0.35/0.015

ROIs according to the ICBM DTI-81 Atlas in the FMRIB library package. For each ROI, the correlation coefficient rho and the 2-sided p-value are specified. Paired ROIs are divided into the right side, left side, and both sides. Most significant correlations in sensible tracts were found in PLIC and corona radiata posterior derived by left-sided N20 (negative correlation in case of FA as indicated). No correlations in sensible tracts were found by right-sided-N20 for FA. ALIC, Anterior limb of internal capsule; PLIC, Posterior limb of internal capsule; CC, Corpus callosum.

*2-sided p-values are not significant *p* > 0.025.

**Table 3:** Shown are the Spearman-Correlation values of N20-latencies from the right and left median nerve for selected ROIs and the DTI-derived parameter Radial Diffusivity (RD).

**ROI**	**Right-side N20—RD (rho/*p*-Value)**	**Left-side N20—RD (rho/*p*-Value)**		
Whole brain	*	0.52/0.0002		
Sensible-tract		0.4/0.006		
Body CC		0.51/0.0003		
Genu CC		0.45/0.002		
Splenium CC		0.43/0.003		
		both	right	left
ALIC		*	*	*
Cerebral Peduncle		*	*	*
PLIC		*	*	0.34/0.02
Corona radiata post.		0.42/0.003	0.42/0.0003	0.39/0.007
Post thalamic radiation		0.46/0.001	0.5/0.0003	0.5/0.0004
Corona radiata sup.		*	*	*
Superior longitudinal fasciculus		*	*	*

ROIs according to the ICBM DTI-81 Atlas in the FMRIB library package. For each ROI, the correlation coefficient rho and the 2-sided p-value are specified. Paired ROIs are divided into the right side, left side, and both sides. Most significant correlations in sensible tracts were found in the corona radiata posterior derived by left-sided N20. No correlations were found by right-sided-N20 for RD. ALIC, Anterior limb of internal capsule; PLIC, Posterior limb of internal capsule; CC, Corpus callosum.

*2-sided p-values are not significant p >0.025.

### Regions of interest

To further investigate distinct anatomical regions, we placed ROIs using the JHU ICBM-DTI 81 -WM-Label-Tract-Atlas as described in the methods section. Significant negative correlations between FA and mCCT were found in the corona radiata posterior (rho −0.48/*p* 0.0007) et superior (rho −0.4/*p* 0.005). Significant negative moderate correlations between FA and mN20 were found in the posterior limb of the internal capsule on the right side (rho −0.33/*p* 0.025). Between RD and mCCT no significant correlations were found. Between RD and left-sided N20 significant moderate correlations were found in the posterior limb of the internal capsule on the left side (rho −0.34/*p* 0.02). Further details of the results are shown in Tables 4, 5.

**Table 4:** Shown are the Spearman-Correlation values of mCCT for selected ROIs and the two DTI-derived Parameters FA and RD.

**mCCT/ROI**	**FA (rho/*p*-value)**			**RD**
Whole Brain	−0.56/0.0005			*
Sensible-Tract	−0.47/0.0009			
Body CC	*			
Genu CC	*			
Splenium CC	−0.37/0.01			
	Both	right	left	
ALIC	*	*	*	
Cerebral Peduncle	*	*	*	
PLIC	*	*	*	
Corona radiata post.	−0.48/0.0007	−0.45/0.001	−0.45/0.001	
Post thalamic radiation	*	*	*	
Corona radiata sup.	−0.4/0.005	*	−0.4/0.005	
Superior longitudinal fasciculus	−0.43/0.002	−0.44/0.002	−0.48/0.0007	

ROIs according to the ICBM DTI-81 Atlas in the FMRIB library package. For each ROI, the 2-sided p-value and rho are specified and divided into the right side, left side, and both sides. Most significant correlations up to p.0007 were found in the Corona radiata posterior for FA (negative correlation as indicated). ALIC, Anterior limb of internal capsule; PLIC, Posterior limb of internal capsule; CC, Corpus callosum.

*2-sided p-values are not significant p >0.025.

**Table 5:** Shown are the Spearman-Correlation values of mN20 for selected ROIs and the two DTI-derived Parameters FA and RD.

**mN20/ROI**	**FA (rho/*p*-value)**			**RD (rho/*p*-value)**		
Whole Brain	−0.54/0.0001			0.51/0.0003		
Sensible-Tract	−0.41/0.004			0.4/0.006		
Body CC	−0.48/0.0008			0.5/0.0004		
Genu CC	−0.42/0.004			0.46/0.001		
Splenium CC	−0.4/0.007			0.45/0.002		
	both	right	left	Both	right	left
ALIC	*	*	*	*	*	*
Cerebral Peduncle	*	*	*	*	*	*
PLIC	*	−0.33/0.025	*	*	*	*
Corona radiata post.	−0.39/0.008	−0.42/0.004	−0.4/0.006	0.42/0.004	0.42/0.004	0.38/0.009
Post thalamic radiation	−0.4/0.005	−0.34/0.02	−0.46/0.002	0.48/0.0007	0.42/0.003	0.48/0.0007
Corona radiata sup.	−0.36/0.02	−0.36/0.02	−0.35/0.02	*	*	0.38/0.009
Superior longitudinal fasciculus	−0.46/0.001	−0.42/0.003	−0.48/0.0007	*	*	0.34/0.02

ROIs according to the ICBM DTI-81 Atlas in the FMRIB library package. For each ROI, the 2-sided p-value and rho are specified and divided into the right side, left side, and both sides. ALIC, Anterior limb of internal capsule; PLIC, Posterior limb of internal capsule; CC, Corpus callosum.

*2-sided p-values are not significant p >0.025.

### Asymmetry in significant voxels

For mN20, the absolute amount of significant voxels was balanced with a tendency for more voxels on the right hemisphere in FA and RD (10.469 right hemisphere vs. 8.369 left hemisphere for FA). Mean CCT show an asymmetry with emphasis on the left hemisphere in FA (1,729 right vs. 1,935 left). Right- and left-sided N20 latencies showed more significant voxels on the left hemisphere for FA and also for RD in left-sided N20. For right- and left-sided CCT, no significant voxels could be reported. More details are shown in Table 6.

**Table 6:** To quantify asymmetry in right and left hemispheres the absolute amount of significant voxels (*p* < 0.05) is shown, based on different latencies for the GLM-design.

**SSEP**	**FA (number of significant voxels)**		**RD (number of significant voxels)**	
	**Right hemisphere**	**Left hemisphere**	**Right hemisphere**	**Left hemisphere**
N20—mean	10.469	8.369	12.345	12.014
N20—right-side	479	536	0	0
N20—left-side	12.833	13.970	15.194	16.178
CCT—mean	1,729	1,935	0	0
CCT—right-side	0	0	0	0
CCT—left-Side	0	33	0	0

More significant voxels were found in RD compared to FA in left-sided N20 with more voxels in the left hemisphere. A tendency for more voxels in the left hemisphere was found in right- and left-sided N20, but not for meanN20. In mean CCT more voxels for FA in the left hemisphere were found. No significant voxels for RD in CCT latencies were found.

When only focusing on sensory ROIs, a tendency for more voxels on the opposite hemisphere in right- and left-sided N20 latencies for FA could be reported. More details are shown in Table 7.

**Table 7:** Shown are numbers of significant voxels (*p* < 0.05) of the sensory-tract-ROI (Corona radiata posterior et superior, posterior limb of internal capsule, cerebral peduncles) for FA and RD based on different latencies of the GLM-design.

**Sensible tract**	**FA (number of significant voxels)**		**RD (number of significant voxels)**	
	**Right hemisphere**	**Left hemisphere**	**Right hemisphere**	**Left hemisphere**
N20—mean	948	719	843	837
N20—right-side	0	4	0	0
N20—left-side	1.366	920	1.193	1.153
CCT—mean	205	393	0	0
CCT—right-side	0	0	0	0
CCT—left-side	0	0	0	0

MeanN20 is balanced, Whereas, right- and left-sided N20 tend to show more voxels in FA and RD on the opposite hemisphere.

## Discussion

We investigated the relationship of somatosensory evoked potential latencies with measures of brain microstructure evaluated by magnetic resonance DTI, asking if they could serve as a surrogate for white matter damage in patients suffering from MS.

Therefore, correlations of SSEP, especially N20 latencies and CCT, to the MRI-derived DTI parameters FA, RD, and AD were analyzed. We found significant negative correlations with FA (as a marker of microstructural integrity) and significant positive correlations with RD (as a marker of demyelination) in widespread regions of the brain exceeding the somatosensory system and involving areas of normal-appearing white matter for mN20. For mCCT, only a significant negative correlation to FA was found. Additionally, we found considerable side differences in N20 latencies for the right and left side, such that left-sided N20 correlated more strongly and in more regions with FA and RD compared to mN20 and right-sided N20. Furthermore, regions in the opposite hemisphere correlated stronger to both left- and right-sided N20. No correlations between SSEP and AD (as a marker of axonal integrity) were detected.

Fractional anisotropy is a quantitative marker for the direction-dependent diffusivity of water molecules in white matter tracts. Our findings of correlations to FA are similar to earlier studies in cases with MS that assessed clinical scores and behavioral measurements (15–17). Asaf et al. found that a relevant decrease of FA occurs in an early stage of RRMS within 3 months of the first onset of symptoms in multiple WM tracts (18). Huang et al. showed a widespread decrease of FA in MS compared to normal controls and patients with CIS (19). Schneider et al. performed a 2-year follow-up DTI measurement and found an early decrease of FA, especially in supratentorial WM tracts (20).

In line with recent studies, we found positive correlations of RD with SSEPs in similar tracts as the negative correlations of SSEP with FA. RD was previously described as a marker of overall tissue integrity in MS lesions, and it might serve as a surrogate for demyelination (21). RD alterations seem to occur mainly in the later course of MS and not in the early stage of the disease (18, 20). In contrast to RD, SSEP latencies displayed no correlations with AD. An explanation could be that prolonged somatosensory evoked potentials are primarily due to demyelination, which is mainly reflected by RD, and not to axonal damage (21, 22). This seems adequate, because myelination is the prerequisite of saltatory propagation of the action potential along the axon, and therefore of conduction velocity.

Electrophysiological examinations employing evoked potentials (EP) are a mainstay in the clinical evaluation and treatment monitoring of patients suffering from MS. Commonly, tests consisting of multiple EPs are used (23–25). Among them, somatosensory evoked potentials in a multimodal approach were shown to be helpful to monitor disease progression and have recently been discussed as a valuable tool for disease monitoring in multicenter studies (26). They can, e.g., be used to assess disease severity since correlations to clinical scores like EDSS were demonstrated (27). Experimentally, it could be shown that SSEPs have close correlations to histologically proven demyelination in a mouse model and can therefore reflect changes in myelination very well, which included for example the monitoring of recurrence to normal SSEPS in case of remyelination in that model (22).

Therefore, being a valuable clinical tool to assess myelination changes in patients with MS, the correlation of whole brain analysis with highly sensitive imaging such as DTI for multiregional microstructural brain assessment seemed warranted. The widespread correlations indicate that the changes which are measurable by DTI and SSEP are not limited to the predefined somatosensory regions and that SSEP latencies appear representative of the extent of demyelination of the whole brain.

In general, the correlations of DTI findings to mN20 showed results of high significance in more regions and clusters compared to correlations of DTI to mCCT latencies (refer to Figure 2). Whereas, mCCT latencies correlate stronger to sensory tracts in FA (refer to Table 4). A reason might be, that longer latencies of somatosensory potentials that includes the spinal cord lead to a more significant correlation with DTI parameters in widespread regions. It is known for SSEP that the longer the measured pathway is the more sensitive it is to detect abnormalities (27). For example, two studies showed that tibial SEPs are more sensitive compared to median SEPs and even to VEPs to detect MS and explain that with the longer sensory pathway (28, 29). Schläger et al., therefore, recommend using tibial SEP and motor evoked potentials (MEP) for monitoring MS because of their long spinal cord component (30). In synopsis to the current literature, it seems to be important to use SSEPs that also involve spinal cord pathways for measuring changes that occur in diseases like multiple sclerosis, since this might carry importance for clinical disability markers as well (31). This known characteristic of SSEP and MEP measurements could explain our findings of different correlation patterns for mN20 and mCCT.

For our additional regions of interest (ROI) analysis, we focused on regions that belong to sensory pathways. In the posterior limb of the internal capsule (IC), we found a less significant correlation to FA and RD and no correlations to FA and RD in the anterior limb. The anterior limb of IC is known for a high density of motor fibers, Whereas, the posterior limb contains predominantly sensory fibers of the lemniscal systems (32). In our study, the region with the highest correlation up to *p* < 0.004 that is part of the somatosensory system was found in the posterior corona radiata (PCR), as defined by the JHU ICBM-DTI 81 -WM-Label-Tract-Atlas (13). The results for mN20 as well as mCCT latencies showed strong correlations in this region. There are few studies examining the meaning of PCR in MS. Anatomically, sensible fibers from Gyrus post-centralis and motor fibers from Gyrus praecentralis ascend and descend in PCR (32, 33). Song et al. showed that the cortico-spinal tract might be located in the PCR and the anterior part of the posterior limb of the internal capsule (31). Various DTI and TBSS studies observed findings in the PCR in MS and showed affections of FA and RD in these areas in the early stage of MS (15, 18, 19). Furthermore, the PCR is among the anatomical structures that were shown to be affected by microstructural changes related to cognitive impairment in patients with MS (34). Our strong correlations in the PCR seem to underline the importance of SSEP as a marker for early microstructural changes in MS. It emphasizes the focus on the somatosensory system, but might also be an indicator of interaction with MS-induced damage in a more complicated manner, exceeding the strict boundaries of the somatosensory system.

Another phenomenon that we cannot explain in detail is the finding of a pronounced difference in the right- and left-sided N20 in their correlation to FA and RD. These findings are suggestive for right- and left-sided CCT but did not reach significance. We observed more significant voxels with higher *p*-values in contralateral regions to the latencies. The interside variability is not pathologic for the total cohort so this cannot serve as an explanation for our observation. It rather seems that even SSEP latencies that are within the reference values (as applies to the majority of our cohort) correlate to the brain microstructure. It might be that handedness of subjects, with a much more common dominance of the right hand, influences this result, but this can only be guessed.

## Limitations

Some limitations must be discussed. One is the retrospective design, even though data were acquired prospectively and in a standardized way. Second, our cohort was relatively small. Third, disease duration could only be reported for 36 subjects. We corrected the GLM model for age, gender, and EDSS but not for disease duration, which could have an effect on the results. Fourth, latencies of N20 or CCT were within the normal range in the majority of subjects, and the findings might be more pronounced in a cohort consisting of a wider variance of the electrophysiological input variables. Another limitation might be the limited spatial resolution of the DTI acquisition of 1.8 × 1.8 × 5 mm. Last is the missing of a healthy control group to compare the microstructural damage with regions of significant correlations.

## Conclusion

Highly significant correlations of FA and RD to SSEP latencies in patients suffering from MS demonstrated that widespread microstructural white matter change, reaching beyond the classic somatosensory regions, is present in MS, at least primarily due to demyelination, and can be estimated by standard clinical SSEP. This underlines the systemic aspect of the disease and points to the usefulness of SSEPs as a non-invasive tool to assess neuroprotective or neuroregenerative approaches in clinical trials.

## Data availability statement

The raw data supporting the conclusions of this article will be made available by the authors, if compatible with data security and safety.

## Ethics statement

The studies involving human participants were reviewed and approved by Ethik-Komission an der Medizinischen Falkutät der Universität Leipzig. The patients/participants provided their written informed consent to participate in this study.

## Author contributions

JH was responsible for data analysis, FSL-suite operation/programming, design of the study, concept, and writing of the manuscript. DL was responsible for data analysis, data acquisition, design of the study, concept and writing, and careful revision of the manuscript. BE was responsible for data acquisition and design of the study. KH was responsible for design of the study, data analysis, and careful revision of the manuscript. FT was responsible for design of the study and careful revision of the manuscript. All authors contributed to the article and approved the submitted version.

## Conflict of interest

The authors declare that the research was conducted in the absence of any commercial or financial relationships that could be construed as a potential conflict of interest.

## Publisher's note

All claims expressed in this article are solely those of the authors and do not necessarily represent those of their affiliated organizations, or those of the publisher, the editors and the reviewers. Any product that may be evaluated in this article, or claim that may be made by its manufacturer, is not guaranteed or endorsed by the publisher.
